# Man With Abdominal Pain

**DOI:** 10.1016/j.acepjo.2025.100089

**Published:** 2025-03-01

**Authors:** Katherine B. Griesmer, Landry Hadderton

**Affiliations:** Department of Emergency Medicine, University of Alabama at Birmingham Heersink School of Medicine, Birmingham, Alabama, USA

**Keywords:** portal venous gas, perforation, procedure, paracentesis, pneumoperitoneum

## 1 Case Presentation

**Figure 1:**
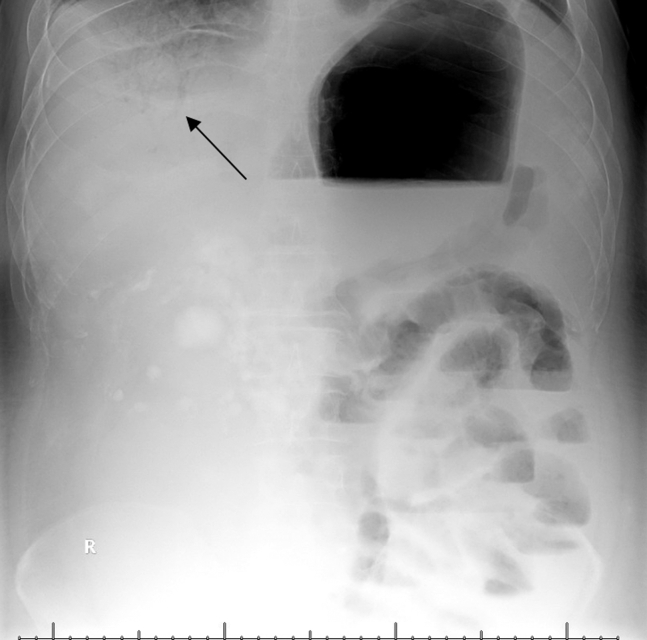
Portal venous gas on portable x-ray (black arrow).

**Figure 2:**
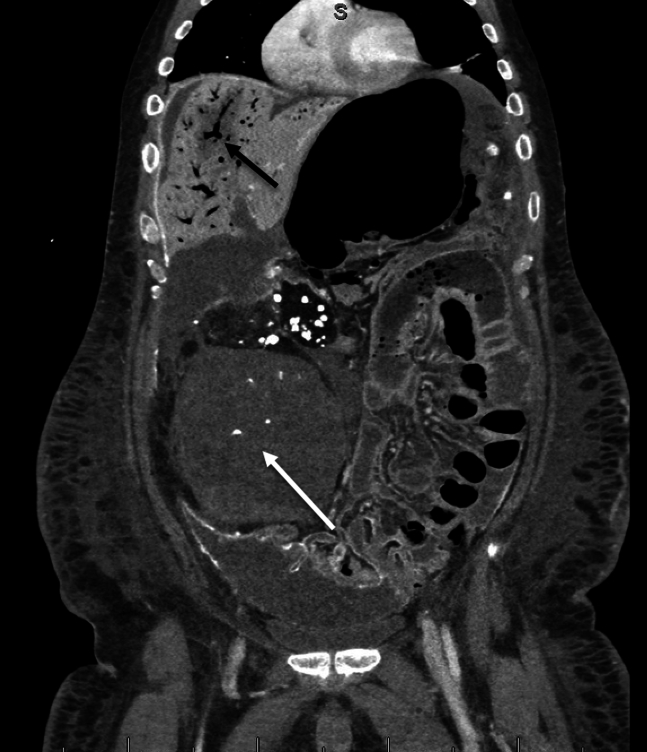
Extensive portal venous gas (black arrow) and small bowel pneumatosis on CTAP. Known retroperitoneal sarcoma is evident as well (white arrow). CTAP, computed tomography abdomen and pelvis.

A 43-year-old man with retroperitoneal dedifferentiated liposarcoma with associated metastatic ascites presented to the emergency department for severe and worsening abdominal pain. He underwent outpatient ultrasound-guided paracentesis earlier that day with over 6 L removed. Upon arrival at the emergency department, the patient presented with severe abdominal pain, indicating an acute abdomen condition. Given the discomfort, an x-ray was utilized for initial imaging (Fig 1). After the x-ray and reduced pain, a computed tomography abdomen and pelvis with contrast was obtained (Fig 2).

## 2 Diagnosis: Portal Venous Gas With Concern for Small Bowel Perforation

Our patient was found to have portal venous gas (Figs 1 and 2), as well as concomitant small bowel pneumatosis, pneumoperitoneum, and concern for possible small bowel infarct and perforation. Although portal venous gas is often connected with neonatal pathology, adults may also display the same rare pathology secondary to bowel ischemia, sepsis, or iatrogenic causes. Previously thought to have a significantly higher mortality rate (75%); given the initial connection with bowel ischemia, it remains an emergent condition with mortality rates of approximately 40%.^1^^,^^2^ Thus, if found on an x-ray, a computed tomography scan should still be performed to rule out bowel ischemia.^1^^,^^2^

Paracentesis, which our patient underwent before presentation, has a rare but known risk of bowel perforation (approximately 0.4% in 1 study) and is found to occur more often in those with advanced liver disease.^3^ With regards to our patient, it is difficult to ascertain if it occurred secondary to his malignancy or recent procedure, with the patient and family electing for comfort care measures given his underlying condition.

## Funding and Support

By *JACEP Open* policy, all authors are required to disclose any and all commercial, financial, and other relationships in any way related to the subject of this article as per ICMJE conflict of interest guidelines (see www.icmje.org). The authors have stated that no such relationships exist.

## Conflict of Interest

All authors have affirmed they have no conflicts of interest to declare.
